# Spin transfer torque driven higher-order propagating spin waves in nano-contact magnetic tunnel junctions

**DOI:** 10.1038/s41467-018-06589-0

**Published:** 2018-10-22

**Authors:** A. Houshang, R. Khymyn, H. Fulara, A. Gangwar, M. Haidar, S. R. Etesami, R. Ferreira, P. P. Freitas, M. Dvornik, R. K. Dumas, J. Åkerman

**Affiliations:** 1 Physics Department, University of Gothenburg, 412 96 Gothenburg, Sweden; 2 NanOsc AB, 164 40 Kista, Sweden; 3 International Iberian Nanotechnology Laboratory, Braga, 4715-330 Portugal; 4 Material Physics, School of Engineering Sciences, Royal Institute of Technology, Electrum 229, 164 40 Kista, Sweden

## Abstract

Short wavelength exchange-dominated propagating spin waves will enable magnonic devices to operate at higher frequencies and higher data transmission rates. While giant magnetoresistance (GMR)-based magnetic nanocontacts are efficient injectors of propagating spin waves, the generated wavelengths are 2.6 times the nano-contact diameter, and the electrical signal strength remains too weak for applications. Here we demonstrate nano-contact-based spin wave generation in magnetic tunnel junctions and observe large-frequency steps consistent with the hitherto ignored possibility of second- and third-order propagating spin waves with wavelengths of 120 and 74 nm, i.e., much smaller than the 150-nm nanocontact. Mutual synchronization is also observed on all three propagating modes. These higher-order propagating spin waves will enable magnonic devices to operate at much higher frequencies and greatly increase their transmission rates and spin wave propagating lengths, both proportional to the much higher group velocity.

## Abstract (web summary)

Nano-contact-based spin wave generation may enable high-frequency magnonic devices but has been limited to long wavelengths and weak signal strengths. Here the authors demonstrate high-order short-wavelength propagating spin waves with increased transmission rates and propagation lengths in magnetic tunnel junction stacks.

## Introduction

Steady-state large-angle magnetization dynamics can be generated via spin-transfer torque (STT)^1–3^ in a class of devices commonly referred to as spin torque nano-oscillators (STNOs)^4–10^. The typical building block of an STNO is a thin-film trilayer stack, where two magnetic layers are separated by a nonmagnetic spacer. The charge current becomes partially spin polarized by the magnetic layers and can act as positive or negative spin wave (SW) damping depending on its polarity. Above a certain critical current density, the negative damping can locally overcome the intrinsic damping, resulting in auto-oscillations on one or more SW modes of the system. To sustain such auto-oscillations, a large current density of the order of 10^6^−10^8^ A/cm^2^ is required, which can be achieved by spatial constriction of the current, e.g. using a nanocontact (NC) on top of a GMR trilayer stack. Such NC-based STNOs are also the most effective SW injectors for miniaturized magnonic devices^11–14^, in particular for short wavelength, exchange-dominated SWs, since the wave vector (*k*) is inversely proportional to the NC radius (*r*_NC_) through the Slonczewski relation *k* = 1.2/*r*_NC_. As the SW group velocity, which governs the data transmission rate, scales with *k*, and the operating frequency with *k*^2^, future ultra-high data rate magnonic devices will have to push the SW wavelength down to a few 10s of nanometers^15^.

**Fig. 1:**
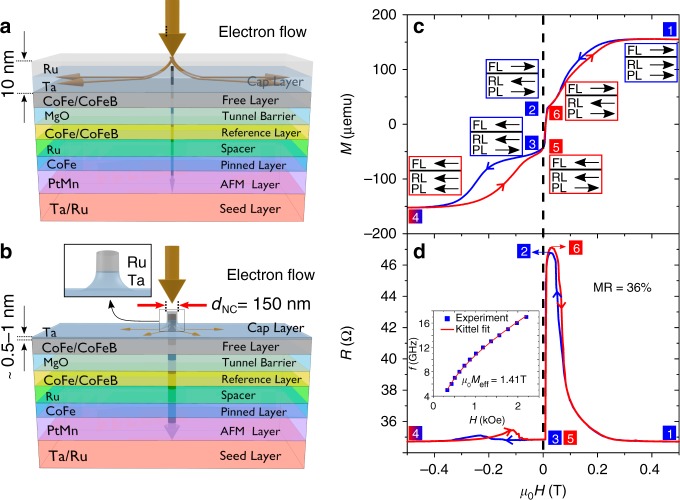
Device schematic, current distribution, and static measurements. **a** Schematic of the material stack showing the current distribution **a** for an ordinary NC and **b** a NC where the Ta/Ru cap has been ion milled into a pillar (inset shows the remaining Ta/Ru). The ion milled pillar reduces the shunt current (orange arrows) in the cap layer and forces a larger fraction of the current to go through the stack (black arrow). **c** Hysteresis loop of the MTJ stack before patterning, with the magnetic field applied along the in-plane easy axis. The magnetic state of the three magnetic layers (free (FL), reference (RL), and pinned layer (PL)) is depicted by the three arrows at six points along the hysteresis loop. **d** The resistance of the final device measured vs. magnetic field along the easy axis showing MR of 36%. Inset in **d** is the frequency of the uniform FMR mode of the free layer as a function of in-plane field. Red solid line is a Kittel fit to extract an effective magnetization of 1.41 T

For efficient electrical SW readout, magnonic devices will also have to be based on magnetic tunnel junctions (MTJs), as tunneling magnetoresistance (TMR) is one or more orders of magnitude higher than GMR^16,17^. The relatively low conductivity of the MTJ tunneling barrier compared with the top metal layers leads to large lateral current shunting for an ordinary NC (Fig. 1a). To force more of the current through the MTJ, we instead fabricate so-called sombrero NCs (Fig. 1b), in which the MTJ cap layer is gradually thinned as it extends away from the NC^18,19^, and use a MgO layer with a low-resistance area (RA) product of 1.5 Ω m^2^ to further promote tunneling through the barrier. The resulting devices exhibit the typical SW modes associated with NC STNOs, such as the spin-wave bullet^20–23^ and the Slonczewski propagating SW mode^3^. In addition, we observe two additional, higher-frequency modes, which we identify as the second- and third-order propagating SW modes mentioned in Ref. ^3^, but never previously observed. We estimate the wavelengths of these two modes to be 120 and 74 nm, i.e., much smaller than the 150-nm nanocontact. Using double nano-contact devices, we furthermore observe mutual synchronization on all three propagating modes, corroborating their propagating character.

## Results

### Magnetostatics

Figure 1c shows the magnetic hysteresis loop of the unpatterned MTJ stack in a magnetic field applied along the in-plane easy axis (EA) of the magnetic layers (for details see Methods). Figure 1d shows the corresponding resistance (*R*) of an MTJ-STNO with a nominal diameter of *d*_NC_ = 150 nm, displaying a magnetoresistance (MR) of 36%, confirming that a significant fraction of the current indeed tunnels through the MgO barrier. The very good agreement between the field dependence of the unpatterned stack and the fully processed MTJ-STNO suggests minimal process-induced changes of the magnetic layers, a strong indication that the free layer (FL) remains intact.

### Magnetodynamics

**Fig. 2:**
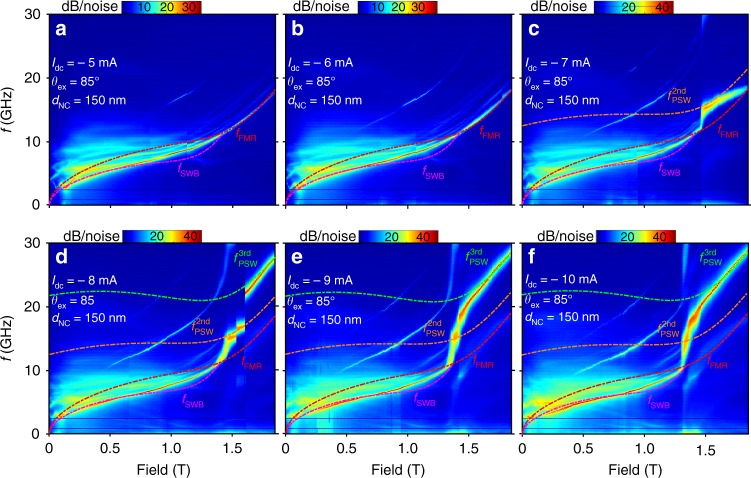
Auto-oscillations vs. field strength: Higher-order Slonczewski modes. **a**–**f** PSD vs. applied field strength (*θ*_ex_ = 85°), and for currents **a** *I*_dc_ = −5 mA, **b** *I*_dc_ = −6 mA, **c** *I*_dc_ = −7 mA, **d** *I*_dc_ = −8 mA, **e** *I*_dc_ = −9 mA, and **f** *I*_dc_ = −10 mA. The pink, red, brown, and green dashed lines represent the calculated frequencies for the SW bullet (*f*_SWB_), the FMR (*f*_FMR_), the second-order Slonczewski mode $\left( {f_{{\mathrm{PSW}}}^{{\mathrm{2nd}}}} \right)$, and the third-order Slonczewski mode $\left( {f_{{\mathrm{PSW}}}^{{\mathrm{3rd}}}} \right)$, respectively

Figure 2 shows the generated power spectral density (PSD) vs. field strength during auto-oscillations at six different drive currents, with the field angle fixed to *θ*_ex_ = 85° (the color plot is assembled from PSD measurements at constant current and field, a few of those shown in Supplementary Figure 2 of Supplementary Information). At the lowest currents, *I*_dc_ = −5 & −6 mA, the strongest mode can be identified as a SW bullet soliton^20–23^. Its frequency, *f*_SWB_ lies well below the ferromagnetic resonance frequency (*f*_FMR_; red dashed line) and can be very well fitted (Eq. 4 in Methods) for fields below 0.7 T. At intermediate fields, 0.7 T < *H*_ex_ < 1.35 T, the bullet signal gradually weakens and its frequency approaches *f*_FMR_, until at 1.35 T it finally disappears as its frequency crosses *f*_FMR_, where self-localization of the bullet is no longer possible. The calculated (Eq. 2 in Methods) internal angle of magnetization, $\theta _{{\mathrm{int}}}^{{\mathrm{crit}}} = 60^\circ$, at the critical field *μ*_0_*H*_ex_ = 1.35 T, is in good agreement with the theoretical prediction^24^ $\theta _{{\mathrm{int}}}^{{\mathrm{crit}}} = 55^\circ$. Above the critical field, we find a weaker mode about 0.2 GHz above *f*_FMR_ consistent with the ordinary Slonczewski propagating SW mode^3^ (see also Supplementary Figure 2 in Supplementary Information).

At stronger negative currents, *I*_dc_ = −7 mA, the PSD in the high-field region changes dramatically, as a much stronger mode appears with a frequency much higher than *f*_FMR_. This change is accompanied by additional low-frequency noise, indicative of mode hopping. Further increasing the negative current strength to *I*_dc_ = −8 mA, first modifies this new mode, after which another sharp jump up to an even higher frequency is observed at about 1.6 T. As we increase the current magnitude further to −9 and −10 mA, this new mode dominates the entire high-field region. The low-frequency noise is now concentrated to the field region just above the critical field, where the 2nd and 3rd modes appear to be competing.

To analyze this behavior, we draw renewed attention to the higher-order propagating SW modes put forward by Slonczewski^3^ but up to this point entirely overlooked in experiments. The excited propagating SWs have a discrete set of possible wave vectors *r*_NC_*k* ≃ 1.2, 4.7, 7.7..., where only the first-order mode (*r*_NC_*k* ≃ 1.2) is discussed in the literature because of its lower threshold current. Taking the literature value^25^ for the free-layer exchange stiffness, *A*_ex_ = 23 × 10^−12^ J/m, and allowing for a reasonable lateral current spread^26^ (an effective NC radius of *r*_NC_ = 90 nm), we find that we can fit the field-dependent frequencies of both the second- and third-mode almost perfectly using the predicted *k* = 4.7/*r*_NC_ and *k* = 7.7/*r*_NC_. The ordinary first-order mode can be equally well fitted (not shown). It is noteworthy that increasingly higher currents are required to excite the higher mode numbers, in agreement with Slonczewski’s original expectations^3^. We also point out that in the original derivation only radial modes were considered, excluding any azimuthal modes. The additional smaller frequency step observed within the 2^nd^ radial mode in Fig. 2(d) could hence be related to a further increase in the exchange energy of a possible azimuthal mode^27^.

**Fig. 3:**
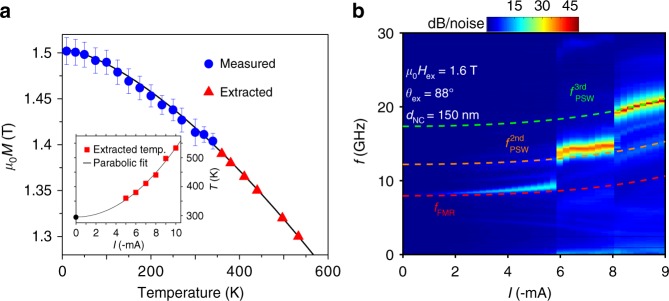
Temperature dependence of the magnetization and current control of the higher-order Slonczewski modes. **a** Temperature dependence of *M*_S_ where the blue data points are CryoFMR measurements on unpatterned areas of the MTJ wafer, the black solid line is a Bloch law fit to these data points, and the red triangles are the extracted values for *M*_S_ from fits to the data in Fig. 2a–f placed on the extrapolated part of the Bloch law. The corresponding temperatures are plotted vs. STNO current in the inset together with a fitted parabola, fixed at room temperature for zero current. The error bars are calculated using the standard error method while fitting the dispersion (*f* vs. H) to Kittle equation in order to extract *M*_S_ (**b**) PSD vs. drive current for another sample with the same nominal size at *θ*_ex_ = 88° and *μ*_0_*H*_ex_ = 1.6 T

In all fits, we allowed *M*_s_ to be a function of *I*_dc_ and used the same *M*_s_ to calculate *f*_FMR_, *f*_SWR_, and $f_{{\mathrm{PSW}}}^{i = 1,2,3}$. This allows us to estimate the amount of heating due to the drive current. Figure 3a shows the variation of *M*_s_ as a function of temperature (blue circles) measured from 10 to 340 K using temperature-dependent FMR spectroscopy on unpatterned areas of the MTJ stack. We can fit *M*_s_(*T*) to a Bloch law function and extrapolate this dependence to higher temperatures (black solid line). The red triangles in Fig. 3a then show the extracted *M*_s_ values at each *I*_dc_ placed on the extrapolated fit, which allow us to extract the local temperature of the FL underneath the NC. As can be seen in the inset, the temperature shows a parabolic rise with increasing current, indicative of Joule heating. The current-induced temperature rise at e.g., *I*_dc_ = −9 mA is about 220 K, which is consistent with literature values of nanoscale temperature gradients in similar structures sustaining similar current densities^28^.

We then show how we can control which propagating mode to excite by varying the current at constant applied field (Fig. 3b). We can again fit the three modes very accurately using the current-dependent *M*_s_(*I*_dc_) extracted from Fig. 3a. The weak current tunability of our MTJ-based NC-STNOs is consistent with the weak non-linearity values found in the literature on MTJ pillars^29,30^ and is advantageous as it reduces any non-linearity driven increase in phase noise from amplitude noise^31,32^. It is also consistent with theoretical predictions that the non-linear frequency shift, at a constant current, should decrease strongly with increasing NC size and only be prominent for sub 100-nm NCs^33^. Since our large MTJ-based STNOs can auto-oscillate at about the same currents as the smaller GMR-based STNOs, we conclude that the generated SWs should have a much weaker nonlinear frequency shift, i.e., should be considered as quasi-linear.

### Mutual synchronization

**Fig. 4:**
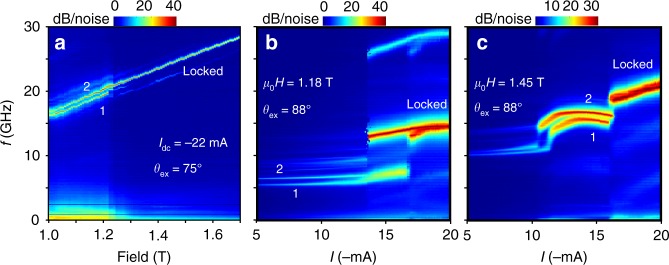
Mutual synchronization on the first, second, and third Slonczewski modes. Mutual synchronization of MTJ-based STNOs having two NCs with a nominal diameter of 150 nm and center-to-center separation of 300 nm. **a** Synchronization on the first Slonczewski mode as a function of external magnetic field *H*_ex_ for a drive current of *I*_dc_ = −22 mA and an out-of-plane field direction of *θ*_ext_ = 75°. Synchronization on **b** the second Slonczewski mode at *H*_ex_ = 1.18 T and **c** the third Slonczewski mode at *H*_ex_ = 1.45 T, as a function of drive current for *θ*_ext_ = 88°. Mutual synchronization on all three Slonczewski SW modes serves as evidence for their propagating nature

In Fig. 4, we show experimentally that it is also possible to achieve spin-wave-mediated mutual synchronization^34–37^ on all three modes, further corroborating their propagating character. Figure 4a shows the PSD vs. field for a double-NC MTJ-STNO auto-oscillating on the first Slonczewski mode (the double NCs were fabricated on the same type of stack as the device above). At fields below 1.22 T, the PSD shows two distinct peaks at high frequency and substantial microwave noise below 2.5 GHz. Above 1.22 T, the two signals instead merge into a single signal with a frequency in between the first two, and the microwave noise disappears. These observations are consistent with highly interacting but individually auto-oscillating regions below 1.22 T and a robust mutually synchronized state above 1.22 T.

As the original double NC devices did not survive the currents required for auto-oscillations on the higher modes, we instead made a second batch of devices based on an improved MTJ stack with a thicker bottom electrode (150 nm CuN instead of 30 nm) to promote more of the current to pass through the tunneling barrier and further reduce the lateral current spread^26^. As a result, we could effectively reduce the threshold current density for all three modes. Figure 4b shows the PSD vs. current from one such optimized double nano-contact device operating at 1.18 T. The NCs first auto-oscillate on the first mode at relatively close frequencies (Fig. 4b). They then jump to the second mode at different currents: one NC at about −13 mA and the other at about −17 mA. As soon as both NCs auto-oscillate on the second mode, their frequencies approach each other, and at about −18 mA, both the first and the second harmonic indicate mutual synchronization. Figure 4c shows the same device at a higher-field of 1.45 T. At this field, the second mode does not show mutual synchronization at any current. However, when both NCs jump to the third mode, their overlapping signals indicate that mutual synchronization on the third mode is also possible.

## Discussion

The possibility of generating higher-order Slonczewski modes has a number of important implications. Their much shorter wavelengths, in our case estimated to 120 nm (2nd mode) and 74 nm (3rd mode), already bring them into the important sub 100 nm range^15^, which only a few years ago was considered out-of-reach for magnonics^38^. As the SW group velocity increases linearly with the wave vector as *v*_gr_ ≃ 4*γA*_ex_*k*/*M*_s_, much faster transmissions can be achieved in magnonic devices and the SWs can travel significantly farther before being damped out. The calculated group velocities for the three observed modes are *v*_1_ = 258 m/s, *v*_2_ = 1010 m/s, and *v*_3_ = 1655 m/s. This is particularly beneficial for mutual synchronization of multiple MTJ-based NCs. For example, one can find the maximum distance of synchronization, *a*_max_, between the two coupled oscillators, using the method developed by Slavin and Tiberkevich^39^. Using typical parameters of coupling strength, ${\mathrm{\Delta }}_{{\mathrm{max}}}/\sqrt {1 + \nu ^2} = 50\,{\mathrm{MHz}}$, and a Gilbert damping of *α*_G_ = 0.015, we find *a*_max_ = 240, 350, and 420 nm for the PSWs with the corresponding frequencies *f*_PSW_ = 13.5, 17.7, and 24.7 GHz observed at *μ*_0_*H*_ext_ = 1.6 T (Fig. 3). As the drive current of both single and mutually synchronized STNOs can be modulated very rapidly^40–45^, high data rate frequency shift keying^46^ will likely be possible using only a small modulation amplitude of the drive current. In addition, novel modulation concepts such as wave vector keying could be readily realized, with possible use in magnonic devices.

We conclude by pointing out that both the nominal NC diameter (150 nm) and the estimated effective NC diameter (180 nm) are much larger than what could be realized using state-of-the-art MTJ lithography. We see no fundamental reason against fabricating NCs down to 30 nm, which would then translate to wavelenghts down to 15 nm and SW frequencies well beyond 300 GHz. The use of higher-order propagating SW modes might therefore be the preferred route toward ultra-high frequency STNOs.

## Methods

### MTJ multilayer

The magnetron-sputtered MTJ stack contains two CoFeB/CoFe layers sandwiching a MgO tunneling barrier with a resistance-area (RA) product of 1.5 Ω μm^2^^47–49^. The top CoFeB/CoFe bilayer acts as the FL and the bottom one as the reference layer (RL). A pinned layer (PL) is made of CoFe, which is separated from the RL by a Ru layer. An antiferromagnetic PtMn layer is located right below the PL. The complete layer sequence is: Ta(3)/CuN(30)/Ta(5)/PtMn(20)/CoFe_30_(2)/Ru(0.85)/CoFe_40_B_20_(2)/CoFe_30_(0.5)/MgO/CoFe_30_(0.5)/CoFe_40_B_20_(1.5)/Ta(3)/Ru(7), with thicknesses in nanometer. For the demonstration of mutual synchronization on the higher spin wave modes, the same stack but with a thicker CuN(150) layer was used.

### Nanocontact fabrication

After stack deposition, 16 μm × 8 μm mesas are defined using photolithography. To make the hybrid NC structure, electron-beam lithography (EBL) with a negative tone resist is used to define nanocontacts with a nominal diameter of 150 nm. The negative tone resist is used as an etching mask in the ion beam etching (IBE) process. Etching of the cap layers in IBE is carefully monitored by in situ secondary ion mass spectroscopy to prevent any damage to the layers underneath the cap. After this step, a structure similar to that shown in Fig. 1b is realized. Following the etching process, 30 nm of SiO_2_ is deposited to provide electrical insulation between the cap and top contact. The remaining negative tone resist acts as a lift-off layer this time. The devices are left in a hot bath of resist remover combined with a high-energy ultrasonic machine for a successful liftoff. In order to provide electrical access to the devices, top contacts are defined using photolithography.

### Static characterization

The static magnetic states throughout key points of the reversal are highlighted as insets in Fig. 1c and d. Decreasing the field from a fully saturated state (1) allows the RL to gradually rotate to be anti-parallel (2) with the PL due to the strong antiferromagnetic coupling (AFC). In going from state 2 → 3, the FL switches rapidly in a relatively small field and once again becomes parallel to the RL, hence a minimum *R* is restored. Note that the FL minor loop is shifted toward positive fields in both Fig. 1c and d, indicating some weak ferromagnetic coupling to the RL. Upon further decreasing the field, the magnetic state moves from 3 → 4, as the PL, working against the strong AFC and weaker exchange bias, slowly switches to be parallel to the RL. In Fig. 1d, we find small increases in *R* when moving from states 3 → 4 and 4 → 5. These can be attributed to minor scissoring of the RL and PL layers due to their strong AFC. As one goes from state 5 → 6 → 1, the FL switches to align with the applied field, followed by the RL switching at high field.

### Ferromagnetic resonance (FMR) measurements

The magnetodynamic properties of the free layer (CoFeB) are determined using an unpatterned thin-film stack. The inset of Fig. 1d shows the extracted FL resonance field from broadband FMR measurements (blue squares), fitted with the standard Kittel equation (red line). From the fit, we extract the values of the gyromagnetic ratio, *γ*/2*π* = 29.7 GHz/T, and effective saturation magnetization, *μ*_0_*M*_eff_ = 1.41 T. Subsequent microwave measurement are performed such that the in-plane component of the field lies along the EA of the MTJ stack. We also study the temperature dependence of the magnetodynamcis at low temperature using a NanOsc Instrument CryoFMR system. The low-temperature measurements are performed between 10 K–340 K. At each temperature, the FMR response was measured at several frequencies over the range 4–16 GHz, where an external magnetic field is applied in the film plane. At each frequency, the resonance field (*H*_res_) is extracted by fitting the FMR to a Lorentzian function. We extracted the effective magnetization (*M*_eff_) of CoFeB thin films by fitting the dispersion relation (frequency vs. field) to the Kittel equation $f = \frac{{\gamma \mu _0}}{{2\pi }}\sqrt {H(H + M_{{\mathrm{eff}}})}$, where $\frac{\gamma }{{2\pi }}$ is the gyromagnetic ratio. We fit the variation of *M*_eff_ with the temperature to a Bloch's law to extract *M*_eff_ at higher temperatures (*T* > 340 K).

### Microwave measurements

All measurements were performed at room temperature. In-plane magnetization hysteresis loops of the blanket MTJ multilayer film stacks were measured using an alternating gradient magnetometer (AGM). The MR was measured using a custom-built four-point probe station. The magnetodynamic properties of the unpatterned FL were determined from using a NanOsc Instruments PhaseFMR spectrometer.

Microwave measurements were performed using a probe station with a permanent magnet Halbach array producing a uniform and rotatable out-of-plane field with a fixed magnitude *μ*_0_*H* = 0.965 T. A direct electric current, *I*_dc_, was applied to the devices through a bias tee, and the resulting magnetodynamic response was first amplified using a low-noise amplifier and then measured electrically using a 40 GHz spectrum analyzer (see Supplementary Information). Microwave measurements at higher fields were performed using another custom-built setup capable of providing a uniform magnetic field of up to *μ*_0_*H* = 1.8 T.

### Bullet frequency and PSW spectrum calculation

The angular dependence of the nonlinear frequency coefficient, *N*, is calculated from the following expression^24^:

1 $$N = \frac{{f_{\mathrm{H}}f_{\mathrm{M}}}}{{f_{{\mathrm{FMR}}}}}\left( {\frac{{3f_{\mathrm{H}}^2{\mathrm{sin}}\theta _{{\mathrm{int}}}^2}}{{f_{{\mathrm{FMR}}}^2}} - 1} \right),$$

where *f*_FMR_ is the FMR frequency. $f_{\mathrm{H}} = \frac{\gamma }{{2\pi }}\mu _0H_{{\mathrm{int}}}$, $f_{\mathrm{M}} = \frac{\gamma }{{2\pi }}\mu _0M_{\mathrm{s}}$ and finally *H*_int_ and *θ*_int_ are the internal magnetic field magnitudes and out-of-plane angles, respectively. *H*_int_ and *θ*_int_ are extracted using a magnetostatic approximation:

2 $$H_{{\mathrm{ex}}}\,{\mathrm{cos}}\theta _{{\mathrm{ex}}} = H_{{\mathrm{int}}}\,{\mathrm{cos}}\theta _{{\mathrm{int}}},$$

3 $$H_{{\mathrm{ex}}}\,{\mathrm{sin}}\theta _{{\mathrm{ex}}} = (H_{{\mathrm{int}}} + M_{\mathrm{s}})\,{\mathrm{sin}}\theta _{{\mathrm{int}}}.$$

The frequency of the Slavin–Tiberkevich bullet mode is calculated from^20^:

4 $$f_{{\mathrm{SWB}}} = f_{{\mathrm{FMR}}} + NB_0^2,$$

where *f*_SWB_ is the bullet angular frequency and *B*_0_ is the characteristic spin-wave amplitude. The calculated *f*_SWB_ quantitatively describes the measured field dependence by setting *B*_0_ = 0.46, providing further evidence that this mode is, in fact, a solitonic bullet. The value of *B*_0_ is calculated according to Tyberkevych et al.^20^ and reaches *B*_0_ = 0.46, which is the upper limit of the theory. The spectrum of the propagating spin waves in the linear limit is defined as^50^

5 $$f_0(k) = \frac{{\gamma \mu _0}}{{2\pi }}\sqrt {(H_{{\mathrm{int}}} + Dk^2)(H_{{\mathrm{int}}} + M_{{\mathrm{eff}}}{\mathrm{cos}}\theta _{{\mathrm{int}}} + Dk^2)} ,$$

where *D* = 2*A*_ex_/(*μ*_0_*M*_eff_) is the dispersion coefficient and *A*_ex_ is the exchange stiffness constant.

## Electronic supplementary material

Supplementary Information

## Data Availability

The data that support the findings of this study are available from the corresponding author upon reasonable request.
