# Supplementary material for: Spin transfer torque driven higher-order propagating spin waves in nano-contact magnetic tunnel junctions
Source: Nat Commun. 2018 Oct 22;9:4374. doi: 10.1038/s41467-018-06589-0 (PMC6197248; doi:10.1038/s41467-018-06589-0)
Supplement: Supplementary file 1 — Supplementary Information [file 41467_2018_6589_MOESM1_ESM.pdf]

Supplementary Information

Spin transfer torque driven high-order propagating spin waves in nano-contact magnetic tunnel junctions

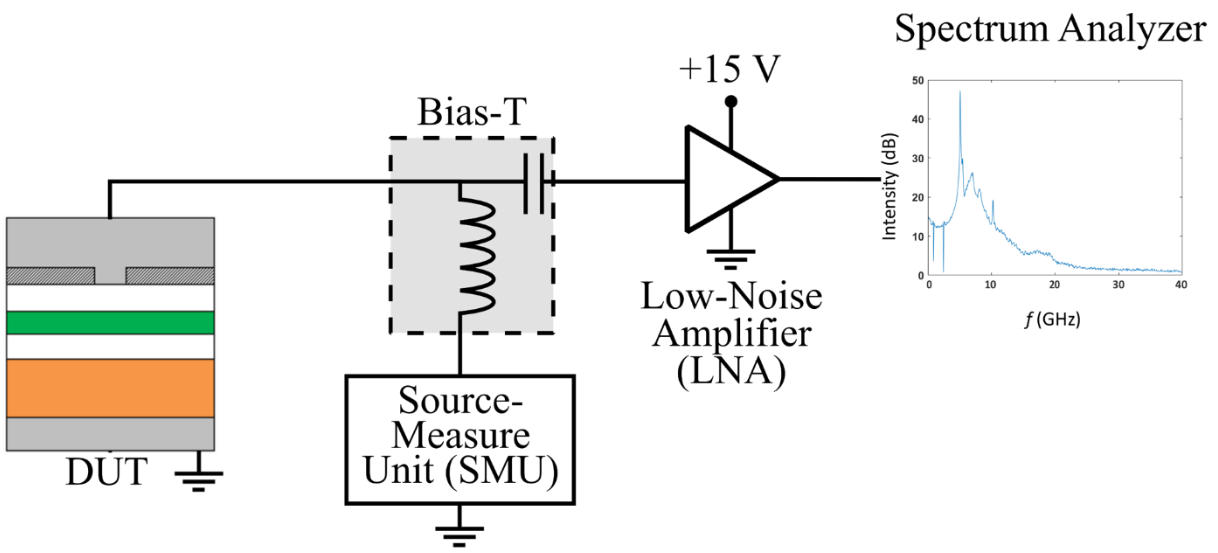

Supplementary Figure 1. Schematic of the measurement set-up. The device under test (DUT) is contacted by a ground-signal-ground probe which applies the dc current and picks up the response of the device. The response is a amplified by a low noise amplifier before going to the spectrum analyzer.

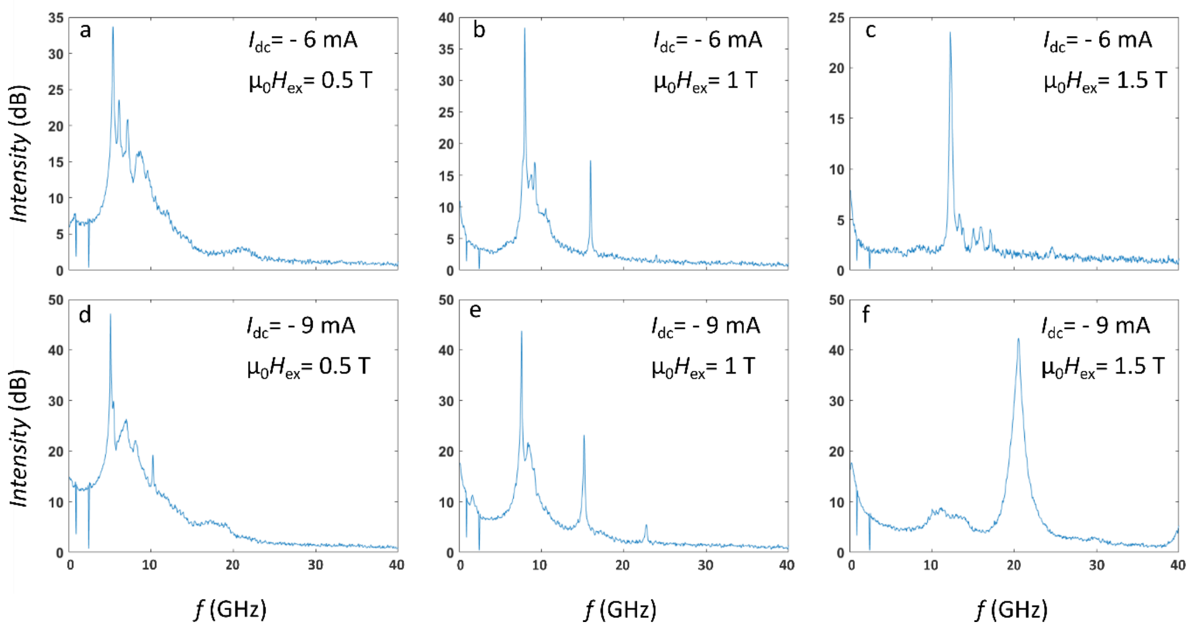

Supplementary Figure 2. PSD examples. Six different spectrum analyzer measurements of the power spectral density from one of the measured MTJ-STNOs in the study. The PSD is plotted on a logarithmic scale to also bring out the much weaker modes. A collection of many such measurements are then assembled to make the color plots in Fig.2.

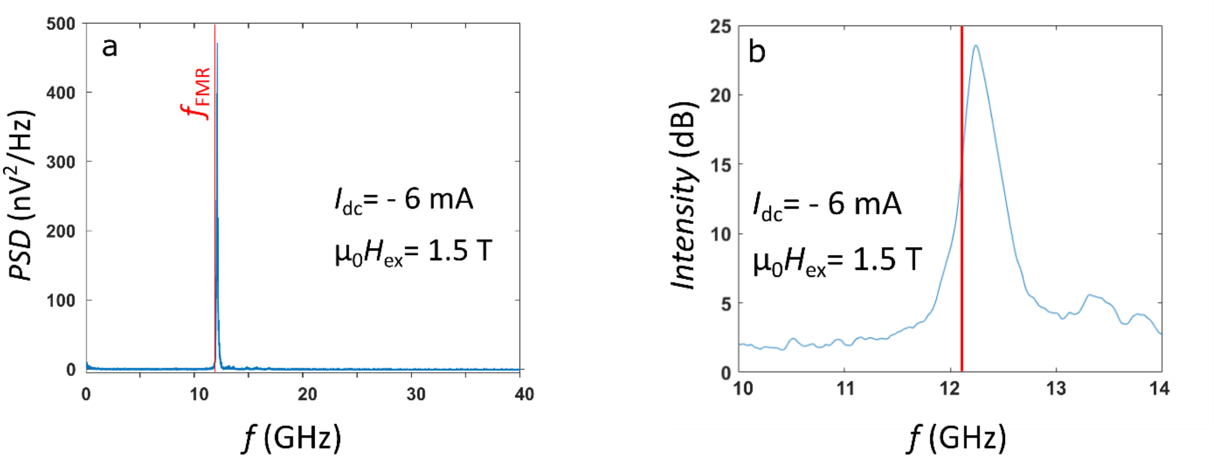

Supplementary Figure 3. Ordinary first-order Slonczewski mode. a) Full spectrum analyzer measurement of the output from the MTJ-STNO plotted on a linear scale. The spectrum is dominated by a single mode located approximately 200 MHz above the FMR frequency. b) A zoom-in on the same signal, now plotted on a logarithmic scale.
